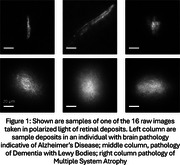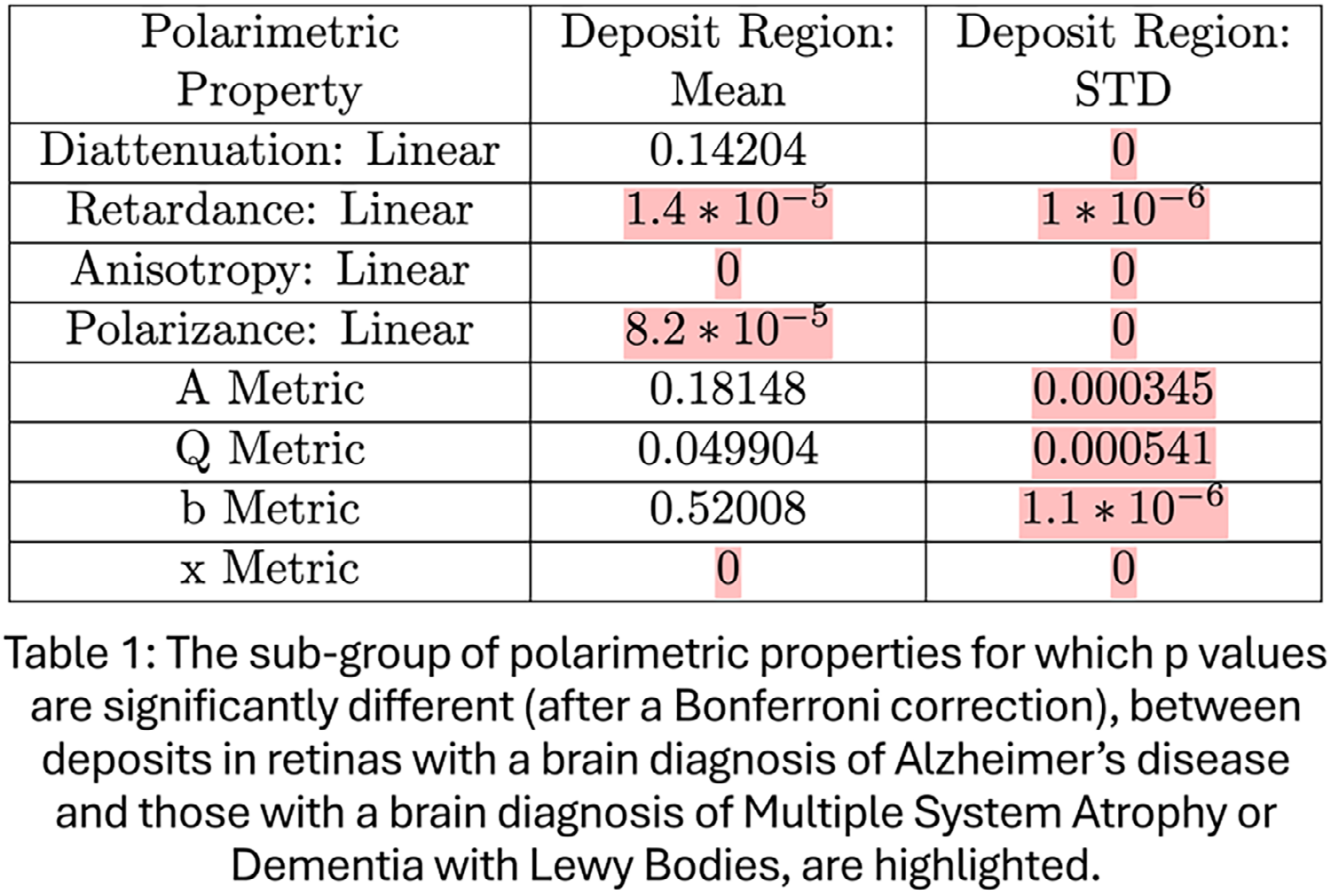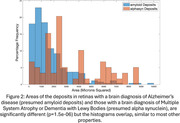# Retinal imaging in polarized light of two different protein deposit types found in the retina in different neurodegeneration diseases (NDDs)

**DOI:** 10.1002/alz.089149

**Published:** 2025-01-09

**Authors:** Melanie CW Campbell, Hannah Gallop, Erik L Mason, Laura Emptage, Rachel Redekop, Monika Kitor, Ging‐Yuek Robin Hsiung, Ian R MacKenzie, Veronica Hirsch‐Reinshagen

**Affiliations:** ^1^ University of Waterloo, Waterloo, ON Canada; ^2^ Vancouver Coastal Health Research Institute, Vancouver, BC Canada; ^3^ The University of British Columbia, Vancouver, BC Canada; ^4^ Vancouver General Hospital, Vancouver, BC Canada

## Abstract

**Background:**

Amyloid and alpha synuclein proteins are brain biomarkers of different neurodegenerative diseases, many years before symptoms. We have shown that imaging dye‐free with polarized light makes retinal amyloid deposits visible as a biomarker, in the brain, of amyloid and Alzheimer's disease (AD) severity. Here, we extend to presumed retinal alpha synuclein deposits in those with brain pathology consistent with alpha synuclein. We have previously shown, using machine learning, that polarized light interactions of pure amyloid beta deposits and pure alpha synuclein deposits, grown on glass, differ.

**Methods:**

Eyes and brains were donated by 10 individuals with brain pathology consistent with amyloid and moderate to high likelihood of AD, and from two individuals with pathology in which brain alpha synuclein is expected ‐ one with dementia with Lewy bodies (DLB) and one with multiple system atrophy (MSA). Retinas were fixed and flat‐mounted, then imaged in differing polarized light states. 274, 47 and 12 deposits were imaged in retinas of those with AD, DLB and MSA respectively. Interactions with polarized light were calculated for each protein deposit imaged.

**Results:**

Presumed amyloid beta and alpha synuclein deposits were visible in polarized light in retinas with brain pathologies consistent with AD, or DLB and MSA respectively (Figure 1). After correction for repeated measures, the means of many polarized light interactions were significantly different for the two presumed deposit types (Table 1). The distributions of the strengths of polarized light interactions and deposit areas (Figure 2) overlapped across deposit types. However, a combination of polarized light interactions separated the presumed retinal deposits of amyloid from those of alpha synuclein in two machine learning algorithms: random forest with an accuracy of >85% and a convolutional neural network (CNN) with an accuracy of > 90%. Linear retardance, which helped to separate the two types of retinal deposits in CNN was previously used to separate deposits of the two pure proteins.

**Conclusions:**

Multiple interactions with polarized light differ between deposits of different retinal proteins. Dye‐free polarized light imaging of the retina shows promise in differentiating these deposits as biomarkers of different neurodegenerative diseases in the brain.